# Mechanistic Modeling of Intramuscular Administration of a Long-acting Injectable Accounting for Tissue Response At the Depot Site

**DOI:** 10.1208/s12248-025-01171-1

**Published:** 2025-10-24

**Authors:** Daniela A. Silva, Maxime Le Merdy, James Mullin, Nilesh Malavia, Khondoker Alam, Eleftheria Tsakalozou, Abdullah Al Shoyaib, Yan Wang, Diane Burgess, Viera Lukacova

**Affiliations:** 1 Simulations Plus, Inc., Research Triangle Park, PO Box 12317, Durham, NC 27709, USA; 2 Department of Pharmaceutical Sciences, University of Connecticut, Storrs, CT 06269, USA; 3 Office of Research and Standards, Office of Generic Drugs, Center for Drug Evaluation and Research, U.S. Food and Drug Administration, Silver Spring, MD 20993, USA

**Keywords:** intramuscular depot, immune response, long-acting injectables

## Abstract

The tissue response to long-acting injectables (LAIs) suspension injection may impact the product *in vivo* performance. One such response is the formation of an inflammatory cell layer (ICL) resulting in an envelope around the injected particles. This study aims to use a mechanistic model to describe the clinical *in vivo* exposure and performance of an intramuscular LAI suspension and evaluate impact of ICL physiological response at the injection site in humans. Aripiprazole lauroxil (AR-L) was used as the model drug. A baseline pharmacokinetics model was built and validated for aripiprazole. The impact of inflammation on the LAI *in vivo* performance was assessed by including an ICL model. The developed pharmacokinetic model adequately described the observed plasma profiles of AR following intravenous and oral administration in humans. The initial intramuscular predictions assumed that the absorption rate is dependent on the dissolution and partitioning of AR-L into the systemic circulation from the intramuscular (IM) depot. The simulation resulted in a shape mismatch between the simulated and observed data and an earlier predicted T_max._ The inclusion of an ICL in the model resulted in adequate predictions (fold errors less than 25%) of the exposure and shape of the plasma concentration–time profiles. Utilizing a time-dependent change in ICL thickness resulted in reasonable predictions of AR pharmacokinetic profiles following IM administration of multiple strengths of the AR-L suspension. This shows the utility of physiologically based pharmacokinetic (PBPK) model in mechanistically describing the *in vivo* performance of LAIs.

## Introduction

Long-acting injectables (LAIs) are designed to release an active pharmaceutical ingredient (API) over an extended period to obtain prolonged therapeutic exposure, thus decreasing drug administration frequency. This, in turn, significantly improves patient compliance compared to regimens that require daily oral administration (1). Reducing the frequency of administration is especially beneficial for patients with conditions such as schizophrenia, alcohol or opioid abuse, where missed doses could lead to behavioral instability or relapses (2). Aripiprazole (AR) is an example of an antipsychotic drug that is available as an intramuscular (IM) LAI in the U.S.A. (3).

Generic development of LAIs has proven challenging due to long development timelines, complex design of bioequivalence studies, complexity of Q1/Q2 requirements “(i.e., qualitative and quantitative sameness to the reference listed drug)”, and limited understanding of tissue response to injected material, impacting *in vivo* performance (2, 4). For example, several studies have indicated that for solid sustained-release depots (e.g., LAI suspension), the inflammatory processes at the injection site are crucial factors affecting both the local tolerability and the systemic drug absorption (5, 6). One such response is the formation of an inflammatory cell layer, known as foreign body reaction (FBR), that develops into a fibrotic response forming an envelope (i.e., immune cell layer; ICL) around the injected particles (5, 7, 8). The dissolved drug will diffuse through the ICL to reach systemic circulation. Consequently, drug diffusion through the ICL may become the limiting step for drug absorption, thus masking the link between *in vitro* measured properties and *in vivo* product performance (6, 9).

In this scenario, mechanistic modeling can be valuable in establishing a link between *in vitro* formulation characteristics and *in vivo* performance by incorporating the physiological response at the depot site (4). For example, local acute inflammatory response to injected foreign material may lead to local oedema, affecting the volume available for dissolution. This can be modeled by including a function changing the depot volume over time, such as described by Silva et. al. (4). The authors reported a better prediction of observed pharmacokinetic (PK) profile after including an inflammation function capturing the faster release observed in the first days post administration (4).

While oedema may result in a faster release rate and appearance in systemic circulation, a fibrotic inflammatory response (ICL) may delay drug appearance in systemic circulation. This study aims to use a mechanistic model to describe the clinical *in vivo* exposure and performance of an IM LAI suspension and evaluate impact of ICL physiological response at the injection site in humans. Aripiprazole lauroxil (AR-L) was used as the model drug. AR-L is a prodrug with a very low solubility resulting in slow dissolution at the depot site. After AR-L dissolution and transfer into systemic circulation, the rapid conversion of AR-L to AR is mediated by systemic esterase cleavage. The enzyme-mediated cleavage of AR-L generates N-hydroxymethyl aripiprazole and lauric acid. Subsequent rapid water-mediated hydrolysis of N-hydroxymethyl aripiprazole yields AR and formaldehyde (10).

## Materials and Methods

### *In vitro* Characterization of Aristada^®^

#### Materials

Aristada^®^ (aripiprazole lauroxil IM extended-release suspension), a reference listed drug product (RLD), was obtained from AmerisourceBergen. Aripiprazole lauroxil, the active pharmaceutical ingredient (API), was obtained from MCE Chemicals^™^. Sodium dodecyl sulfate, phosphate-buffered saline, sodium acetate, Tris buffer, and Tween 20 were purchased from Sigma-Aldrich.

#### Thermal Analysis

##### Thermogravimetric Analysis

Thermogravimetric analysis (TGA) of RLD Aristada^®^, containing aripiprazole lauroxil as the API, was performed using TA Q500 TGA universal instruments (New Castle, DE, USA). Prior to experiments, TGA pans were preheated to temperatures exceeding 500° C to remove all the residuals from previous analysis. For sample preparation, 10 mg of suspension was washed 3 times with 1 ml of deionized water to remove all excipients of suspending media. After the third wash, 1 ml of deionized water was added, and the sample was centrifuged at 13,000 RPM for 5 min to allow separation of aripiprazole lauroxil API. The separated API was then dried in a vacuum oven at 40° C for 24 h. Approximately 2 mg of dried API was loaded into tared TGA pans. The pans were then equilibrated at 25° C and heated up to 400° C at a ramp rate of 10° C/min. Data analysis was performed using TA universal analysis software.

##### Differential Scanning Calorimetry

Differential scanning calorimetry (DSC) of Aristada^®^ was performed using TA Q1000 differential scanning calorimeter (Universal TA instruments, New Castle, DE, USA), which was equipped with a refrigerated cooling system. Approximately 3 mg of dried aripiprazole lauroxil suspension was equilibrated at 30° C followed by the application of heat up to 400° C at the rate of 10° C/min. Nitrogen was used as the purging gas at a flow rate of 50 ml/min throughout the sample run to maintain an inert environment. Data analysis was performed using TA universal analysis software.

#### Solubility Analysis

The aqueous solubility of aripiprazole lauroxil was determined in different aqueous media, including deionized water at different pH. Saturated solutions were prepared by dispersing 1 mg of aripiprazole lauroxil in 1 mL of the selected aqueous medium, followed by incubation in a shaker bath at 37 °C and 100 rpm for 48 h to ensure equilibrium. After incubation, 100 μL of the dispersion was filtered using a Duropore^™^ 96-well filter plate equipped with a hydrophilic PVDF membrane and a pore size of 0.45 μm. The resulting filtrate was analyzed using ultra-performance liquid chromatography (UPLC) to determine the concentration of dissolved aripiprazole lauroxil. All the experiments were performed in triplicate.

The procedure for UPLC analysis was as follows. Separation and analysis of aripiprazole lauroxil was achieved using Alliance Waters UPLC system. The UPLC system is equipped with a quaternary solvent manager (QSM), photodiode array detector (PDA) and sample manager. The data acquisition and analysis were performed using Waters EMPOWER software and the chromatographic separation was achieved using a C18 Acquity BEH column (50 mm X 2.1 mm X 1.7 mm). The samples were eluted by isocratic elution (4 min elution time; 0.4 ml/min flow rate, 100% A and 0% B), using a mobile phase of 10% 0.05 M ammonium acetate and 90% acetonitrile. The optimized parameters for the effective separation are as follows: flow rate of 0.4 mL/min, injection volume of 10μL and column oven temperature of 37°C.

#### Solid state Characterization- PXRD Analysis

The crystallinity of aripiprazole lauroxil API in RLD Aristada^®^ was analyzed using an X-ray diffractometer (Benchtop D2 phaser, Bruker AXS Inc., Madison, WI, USA) equipped with Cu-kα radiation. Scans were performed at the 2θ values ranging from 5° to 40° with the step increment of 0.018°/step and 0.035°/step. The voltage and current were set at 30 kV and 10 mA.

#### Particle Size Analysis

The particle size of all the formulations was characterized using a MasterSizer^™^ 3000 (Malvern Panalytical Inc., MA, USA) with a Hydro MV (medium volume) automated dispersion unit. In brief, about 30 μL of Aristada^®^ was diluted to 1 mL with water. The dispersion was added to the chamber of the dispersion unit with stirring at 2500 rpm. The particle size (volume weighted) and size distribution were recorded at 10% (Dv10), 50% (Dv50), and 90% (Dv90). The SPAN value for size distribution was determined using the formula (Dv90-Dv10)/Dv50. All the measurements were performed in triplicate and the data were presented as mean ± standard deviation.

Additionally, microscopic investigations were conducted to assess the morphological characteristics and particle size of the primary API particles. Polarized light microscopy was employed to observe the particle shape, surface features, and birefringence properties, which can provide indirect evidence of crystallinity.

### Modeling Workflow

**Fig. 1:**
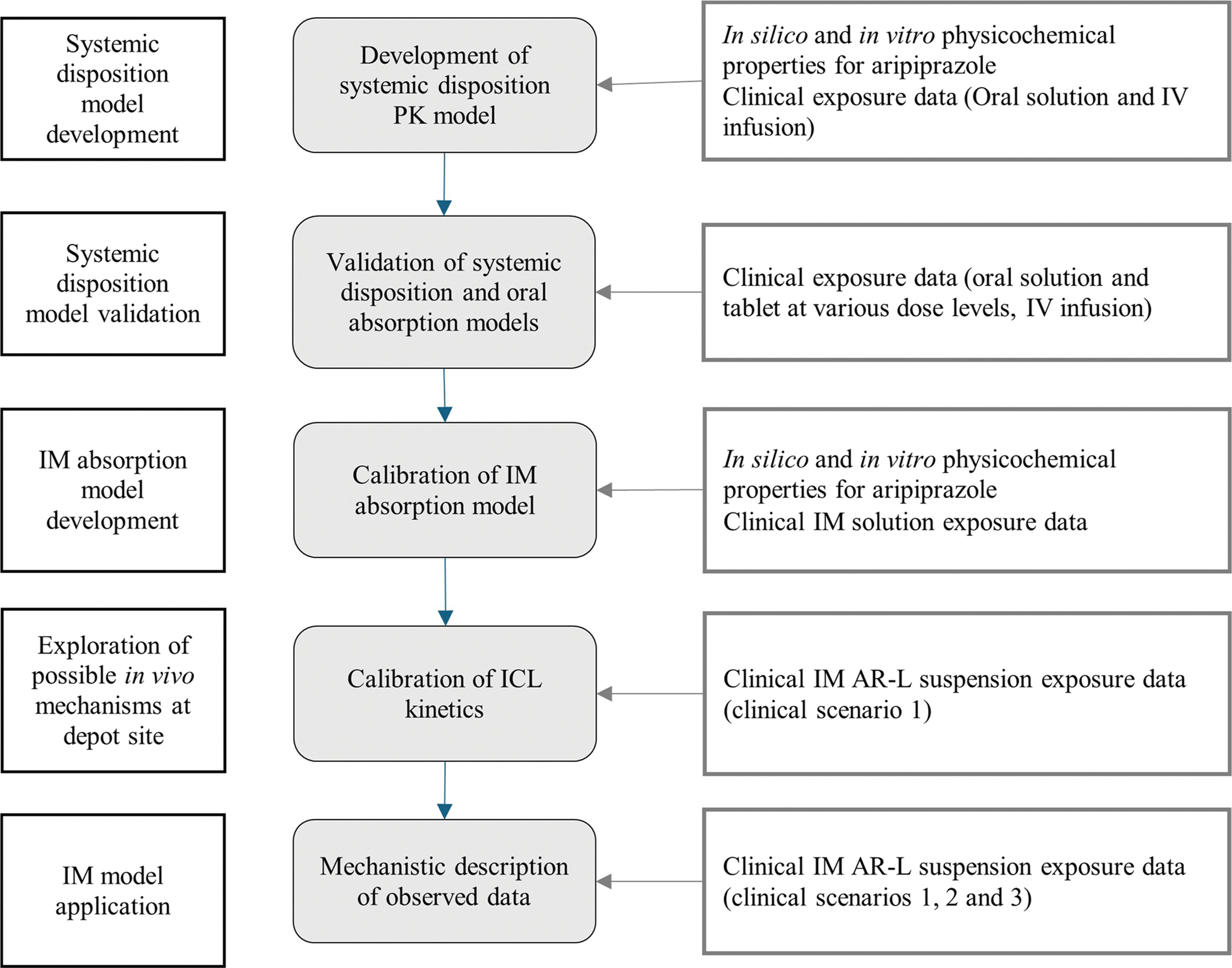
Diagram of modeling workflow

The modeling workflow is shown in Fig. 1 and the data used for each step is summarized in Table I. The baseline PK model was built using measured and predicted physicochemical properties of AR. A compartmental model with linear clearance was built based on intravenous (IV) infusion and oral (PO) solution data for humans. External validation of this IV-PO model was performed by predicting AR PK in multiple clinical scenarios (e.g., oral solution and tablet). Once the external validation was complete, the mechanistic PBPK model describing AR PK following IM administration could be validated. The IM absorption model was validated using clinical PK of AR administered as an IM solution. This validated approach to calculate tissue/plasma partition coefficient was applied to AR-L (more details in Intramuscular Model). Subsequently, two scenarios were tested for the IM AR-L suspension modeling approach. The first one was to predict the plasma concentration profile based on the solubility of the compound in the IM milieu, particle size, depot volume, and tissue/plasma partition coefficient. The second approach accounted for an additional fibrotic inflammatory response.

### Physiologically Based Pharmacokinetic Model

#### Physicochemical Properties & Software

The physicochemical and biopharmaceutical properties for AR and AR-L were defined using a combination of in silico predictions (ADMET Predictor module. 11.2) based on their chemical structures, as well as *in vitro* and *in vivo* data obtained from literature. The PBPK model for AR-L/AR was developed using GastroPlus v. 9.8.3 (Simulations Plus, Inc.). The model input parameters are listed in Table II.

#### Systemic Disposition Model

The built-in PKPlus^™^ module in GastroPlus^®^ was used to fit a compartmental model for AR using oral solution data (15, 20 and 30 mg). A three compartmental model had the best fit according to the calculated R square, Akaike Information Criterion and Schwarz Criterion, hence it was the chosen model to describe AR systemic disposition in humans. Once the PK parameters were fitted across the three oral solution doses, the IV infusion (2 mg) data was used to verify the model (11, 12). This was done because the IV study had a limitation in the later concentrations which fell below the lower limit of detection (1 ng/mL) (12). Even though aripiprazole is a substrate of CYP3A4 and CYP2D6 (14), a linear hepatic clearance was applied to model human data (Table II). Several clinical scenarios under fasted state (AR solution 5 and 10 mg and AR tablet 5, 10, 15 and 30 mg) were predicted to assess the assumption of constant clearance (11). For oral route modeling, the first pass liver extraction was calculated with the well stirred model based on liver clearance, blood to plasma ratio, and liver blood flow. The effect of first pass gut extraction was neglected since the liver extraction was estimated to be less than 4% of the dose reaching portal vein.

The oral absorption model was mechanistically described using the Johnson dissolution model. A particle radius size of 22 μm was used for all oral tablet administrations since no measured particle size data was available. Plasma concentration data across all doses were used to fit the particle size.

#### Intramuscular Model

The validated systemic PK model was subsequently used to simulate AR systemic exposure following IM injection of AR solution or AR-L suspension. For the IM model, the injection site was selected according to the clinical study (i.e. gluteal injection) and the effective depot volume (volume of tissue with extracellular space large enough to accommodate the entire injection volume) was estimated based on the injection volume. The Lukacova (default) method for perfusion-limited tissue was used to calculate the drug’s tissue (muscle) to plasma partition coefficient (Kp) and the default GastroPlus v9.5 method was used to calculate the tissue (muscle) fraction unbound (f_ut_) (18). Data from an AR IM solution administration (12) was used to verify the IM model settings.

Subsequently, the established systemic PK and IM model for AR was used to simulate AR exposure following IM administration of Aristada^®^ (AR-L suspension) at three dose levels (150, 300, and 400 mg AR equivalent) (11). The *in vivo* dissolution of AR-L was simulated using the Johnson dissolution model with measured particle size and the diffusion layer thickness was fitted to *in vivo* data. Absorption of dissolved AR-L from the depot compartment to systemic circulation was modeled the same way as validated for AR absorption after AR solution IM injection: the IM Kp and f_ut_ values for AR-L were calculated using the default methods based on AR-L physico-chemical properties. The Aristada IM administration simulations assumed total enzymatic cleavage of AR-L to AR as soon as AR-L reaches systemic circulation (19) and the systemic disposition parameters were reflective of AR.

The IM administration of solid material is known to cause an inflammatory response where macrophages fuse forming a multinucleated giant cell that may develop into a fibrotic response forming an envelope around the depot (5). As the inflammation subsides, the envelope becomes thinner until the inflammatory process is resolved (14). This *in vivo* process was explored in the model by including an immune cell layer (ICL) that encapsulates the depot. The time-dependent change in the ICL thickness was fitted to the observed PK data for the 400 mg equivalent IM Aristada dose (11) according to Eq. 1 (5). Then, the same kinetics of ICL thickness was used to simulate the remaining IM Aristada doses for validation. The AR-L diffusion through the ICL and the fraction unbound in the ICL were estimated from AR-L LogP (Eqs. 2 and 3, respectively) (18). The approach taken to estimate ICL surface area was based on the injected dose, assuming a spherical depot shape according to Eq. 4.

(1) $$ICL_{\mathrm{thickness}}=A\times t\times e^{{(}^{-B\times t})}$$

where A and B are user defined constants to account for the ICL kinetics.

(2) $${\mathrm{Diffusivity}}_{ICL}=logD(7.4)>3=10^{-5.9514}$$

(3) $${\mathrm{Fraction}\;\mathrm{unbound}}_{ICL}=\frac{1}{2.12\;\times \;e^{0.523\times logD(7.4)}}$$

(4) $$\mathrm{Surface}\;\mathrm{Area}=4\pi {\left(\frac{3\;\times \;\left(\frac{\mathrm{Dose}}{\rho }\right)}{4\pi }\right)}^{2/3}$$

where ρ is density.

#### Acceptance Criteria for Model Validation

The predictions were considered acceptable when the shape of the simulated plasma concentration vs. time (Cp-time) profile closely matched with the shape of the average observed Cp-time profile by visual inspection and the predicted C_max_ and AUC_0-t_ were within 25% of the observed values.

#### Parameter Sensitivity Analysis

The sensitivity of the model for some parameters was assessed via a parameter sensitivity analysis (PSA) before the inclusion of ICL to assess if other parameters could explain the observed data. These parameters and respective ranges included in this PSA were solubility (3E-5—3E-3 mg/mL), particle size (7.9—141.8 μm), diffusion layer thickness (30—150 μm), intramuscular blood flow (4.8—19.2 mL/min/100 g tissue), depot volume (1—27.2 mL) and intramuscular partition coefficient (2.14—8.55).

The sensitivity of the model to ICL kinetics and formulation properties was also assessed via PSA. The ICL Lag time was varied between 0–1500 h, ICL thickness magnitude (ICL-A) between 2.09E-08–0.29 cm/hr, ICL thickness rate (ICL-B) between 0.003–0.07 1/hr and mean particle size between 1.54–75 μm. In both cases, the PSA was performed with the 300 mg AR equivalent gluteal dose.

## Results

### Thermal Analysis

The DSC analysis revealed that the melting point of aripiprazole was approximately 81° C, consistent with values reported in the literature (Supplemental Fig. 1) (20). This suggests that the vehicle used in the preparation of Aristada^®^ does not influence the melting behavior of the API. The DSC thermogram displayed a single sharp endothermic peak corresponding to crystalline aripiprazole, with no additional peaks observed. This indicates that aripiprazole lauroxil does not form hydrates or solvates in the dispersion medium. TGA was performed to quantify residual organic solvent in aripiprazole lauroxil API, as excessive residual solvent could affect solubility and, consequently, dissolution behavior. The residual solvent content was found to be approximately 0.254%, which is considered low (Supplemental Fig. 1) (21).

### Solubility Analysis

Solubility analysis was conducted to evaluate the solubility of aripiprazole lauroxil in various media, as it is a critical parameter in formulation development. It did not exhibit a significant solubilized fraction across different pH conditions, which may be attributed to its extremely low aqueous solubility. In the commercial suspending medium, only a small amount (~ 7 μg/mL) of aripiprazole lauroxil was in the solubilized form, which is negligible compared to the administered dose of 441 mg/1.6 mL. This low solubility suggests a minimal potential for initial burst release *in vivo* for Aristada^®^. The small solubilized fraction may be due to the presence of excipients such as Tween 80 and polyethylene glycol (PEG) in the dispersion medium. Additionally, solubility studies were performed using varying concentrations of surfactants in water to assess potential release media. Both Tween 80 and sodium dodecyl sulfate (SDS) demonstrated a concentration-dependent increase in solubility (Supplemental Table 1).

### PXRD Analysis

The PXRD pattern of the dried drug product (Aristada^®^) exhibited distinct sharp peaks, indicating a crystalline nature. The most intense diffraction peak was observed at 2θ ~ 4.9 (Supplemental Fig. 2), suggesting the presence of a highly ordered crystal lattice. Additional characteristic peaks were observed at approximately 10.9°, 15.2°, 17.8°, 18.9°, 20°, 21.5°, 23.4°, and 25.8° (2θ). No significant amorphous halo was detected, indicating the absence of an amorphous phase in the sample analyzed.

### Particle Size Analysis

Particle size is a critical formulation attribute for LAI suspensions, as variations during manufacturing process can significantly impact product performance (22, 23). The mean particle size of Aristada^®^, measured using the MasterSizer^™^, Malvern Instruments, UK, was approximately 28 μm with a calculated SPAN value of 1.38. The cumulative Dv10, Dv50 and Dv90 were 13.87 μm ± 0.88, 28.74 μm ± 0.96, 53.54 μm ± 0.71, respectively (n = 3).

Additionally, microscopy imaging under polarized light revealed that the particles exhibited birefringence, indicating the crystalline nature of aripiprazole lauroxil. The dispersed particles were irregular in shape and lacked defined geometry, which could be attributed to the milling process used during formulation or heterogeneity arising during crystallization. Moreover, some particles appeared to be aggregated, possibly due to hydrophobic interactions (Supplemental Fig. 3).

### Systemic Model

**Fig. 2:**
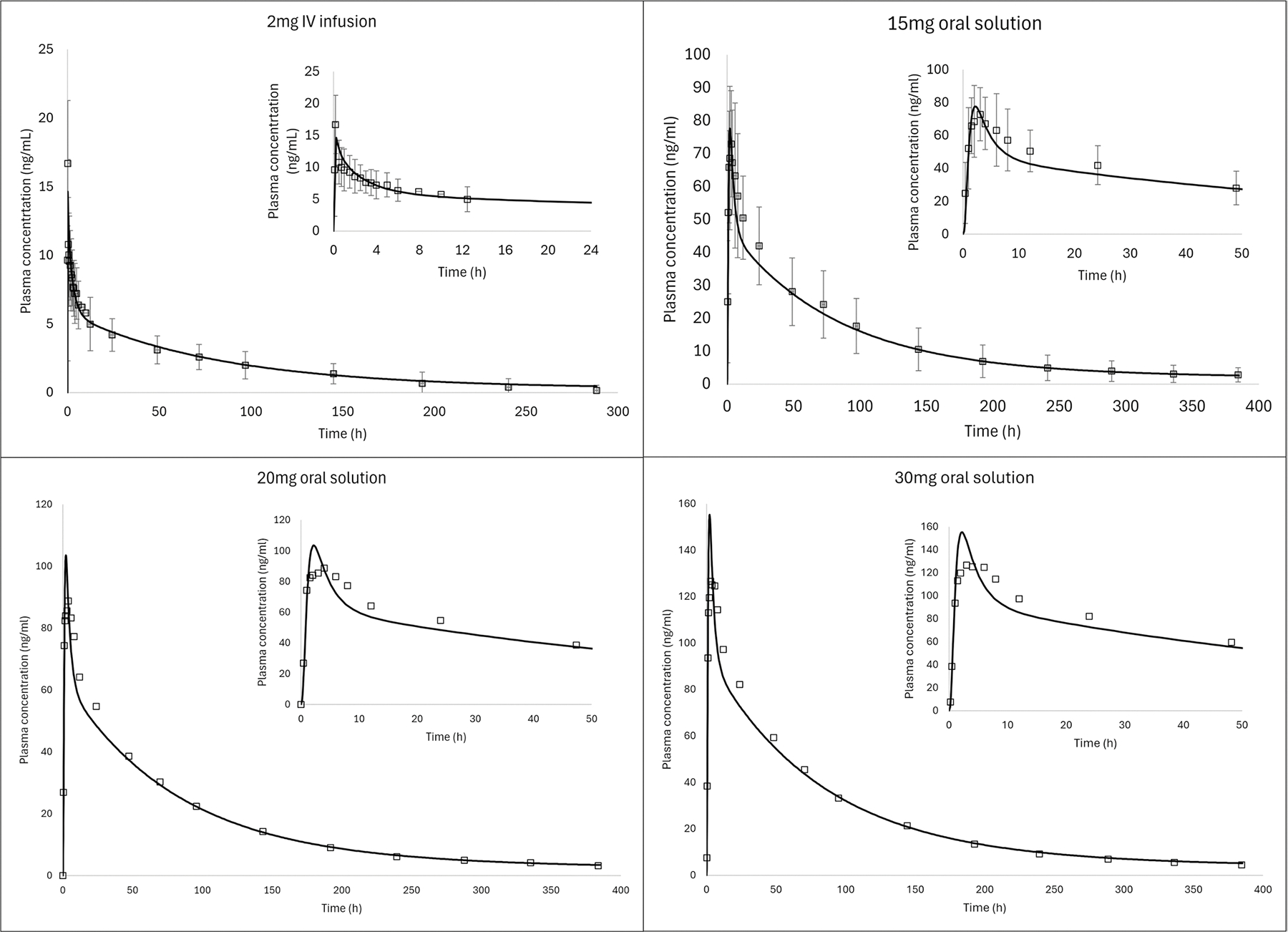
Observed (symbols) and predicted (solid lines) AR plasma concentration vs time profile after intravenous infusion (2 mg) and oral solution (15, 20 and 30 mg) administration of aripiprazole in humans. Dataset used for model development (Table I)

AR plasma concentration data following IV (2 mg) infusion, and oral solution (15, 20 and 30 mg) administration were best described by a three-compartment model. Across the investigated dose range (2 to 30 mg), AR demonstrated a linear systemic PK with a linear hepatic clearance (CL_H_) of 0.03028 L/h/kg; volume of central, second and third compartments of 1.5825L/kg, 2.0831L/kg and 9.1754 L/kg, respectively and constants k_12_: 0.22832 h^−1^, k_21_: 0.17345 h^−1^, k_13_: 0.01108 h^−1^, k_31_: 0.001911 h^−1^. For clinical scenarios used for model development, the simulated/observed ratio ranged from 0.88 to 1.23 for C_max_ and from 0.92 to 1.05 for AUC_0-t_, as shown in Fig. 2 and Table III. The 20 mg and 30 mg solution doses were mispredicted during the absorption phase (first few hours) after dosing, however the distribution/elimination phases were well predicted. Slight absorption misprediction is not impactful for the subsequent use of the model since intestinal absorption is not relevant to the intramuscular model.

**Fig. 3:**
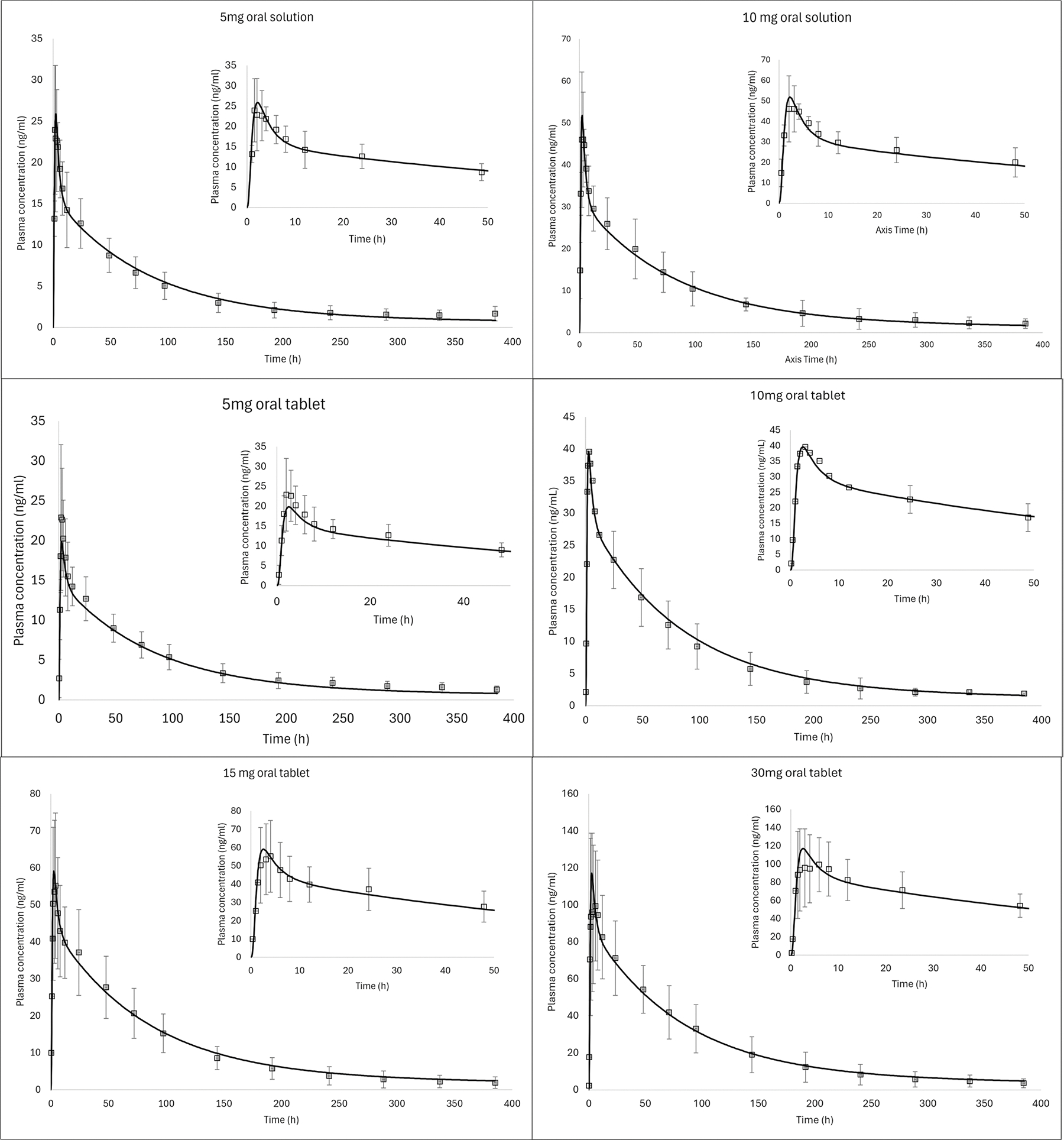
Observed (symbols) and predicted (solid lines) AR plasma concentration vs time profile after oral solution (5, and 10 mg) and oral tablet (5, 10, 15 and 30 mg) administration of aripiprazole in humans. Dataset used for model external validation (Table I)

Data from several other clinical scenarios were used to externally validate the model: oral solution (5 and 10 mg) and oral tablet (5, 10, 15, 30 mg) administrations. The simulated/observed ratio ranged from 0.87 to 1.18 for C_max_ and from 0.88 to 1.04 for AUC_0-t_, as shown in Fig. 3 and Table III. Hence, the developed model adequately described the observed plasma profiles of AR following oral administration in humans with all the simulated C_max_ and AUC_0-t_ values within ± 25% of the observed values (Table III). Based on the simulation results, the systemic distribution and elimination model was deemed acceptable to be used for IM simulations.

### Intramuscular Model

**Fig. 4:**
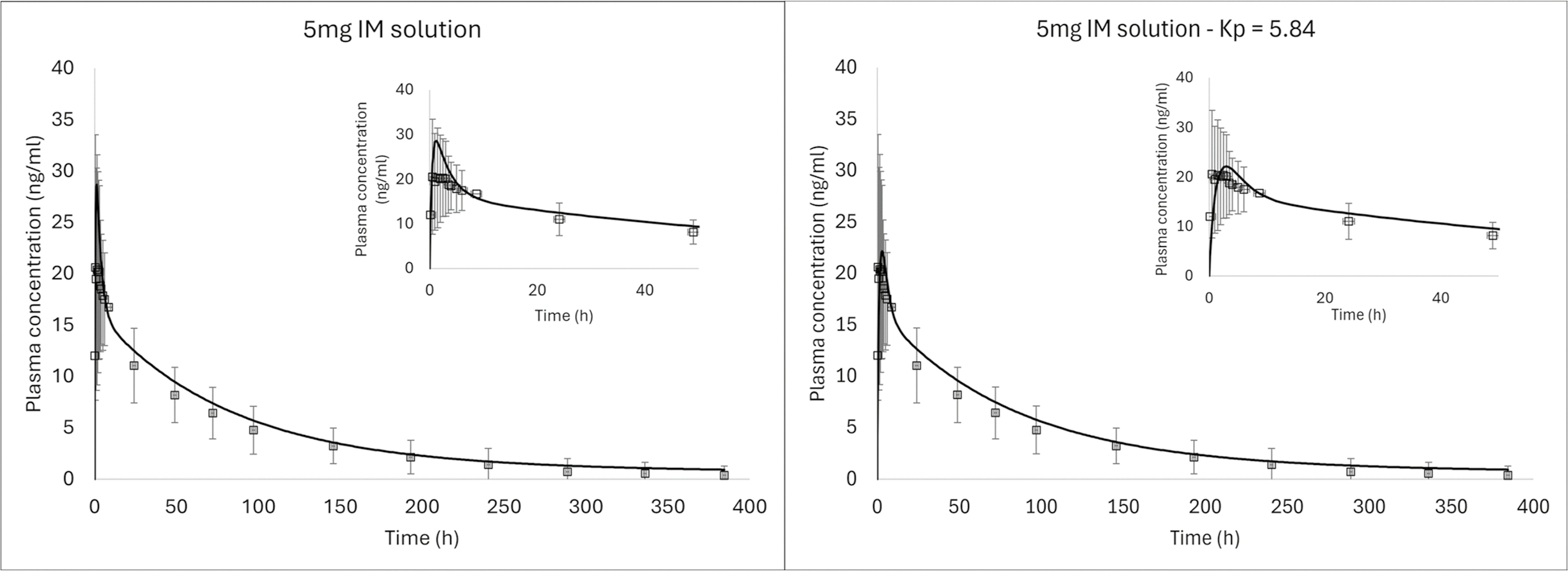
Observed (symbols) and predicted (solid lines) AR plasma concentration vs time profile after IM administration of aripiprazole solution in healthy humans

The calculated IM Kp and f_ut_ for AR were 1.84 and 0.12%, respectively. These parameters were used to simulate AR systemic exposure following its IM solution administration in healthy humans (12). The simulated AUC_0-t_ for IM administration of AR solution was within 25% of the observed value (observed: 1423.2 ng-h/ml; predicted: 1645.3 ng-h/ml; fold-error: 1.16). The fold error on C_max_ was more significant (observed: 20.6 ng/mL; predicted: 28.7 ng/mL; fold-error: 1.39), but within the considerable observed variability (Fig. 4). A possible explanation for the high variability from solution injection may be missed injection site (24), as the IM solution was dosed into the gluteus maximus, i.e. some of the dose could have been dosed to adipose tissue (reported BMI range 18–30 kg/m^2^, mean BMI: 25.5 kg/m^2^) (12). To test this hypothesis, the IM Kp and f_ut_ were adjusted to the median value between the calculated muscle and adipose, i.e. Kps 1.84 and 9.84, respectively, median Kp = 5.84, and f_uts_ 1.20E-03 and 2.24E-04, respectively, median f_ut_ = 0.712E-03. This resulted in better prediction for Cmax (predicted: 22.1 ng/mL; fold-error: 1.07). The reported average of individual Cmax and Tmax were 23.7 ng/mL (CV% 41) and 3 h (range 0.50 h –10 h), respectively. The predictions with both Kps and f_uts_ were within the range of observed values for Cmax and Tmax, which supports the hypothesis of missed injection site.

Given the variability in the observed data during the absorption phase, and the uncertainty as to how much could have been dosed into the subcutaneous space, the approach of calculating IM Kp with the default methods was maintained and the IM Kp for AR was kept at the calculated value of 1.84. The IM absorption model approach, i.e. Kp and f_ut_ default calculation methods, were deemed acceptable to be used for the AR-L IM suspension simulations. Additionally, upon administration of a poorly soluble compound like AR, precipitation can happen at the injection site, which was not accounted for in the model.

The systemic AR PK model was used to simulate AR exposure after IM administration of Aristada (AR-L suspension) at three dose levels (150, 300, and 400 mg AR equivalent). AR-L is synthesized with a proprietary technology (LinkeRx; Alkermes) designed to create a pro-drug from molecules that lack a hydroxyl functional group, such as AR. The modified properties of the pro-drug (AR-L), such as low solubility, result in extended systemic release after injection (10, 19). Such compounds can be formulated as simple aqueous suspensions, and dissolution of AR-L is the rate limiting step in the depot followed by rapid absorption of the pro-drug into systemic circulation (19).

**Fig. 5:**
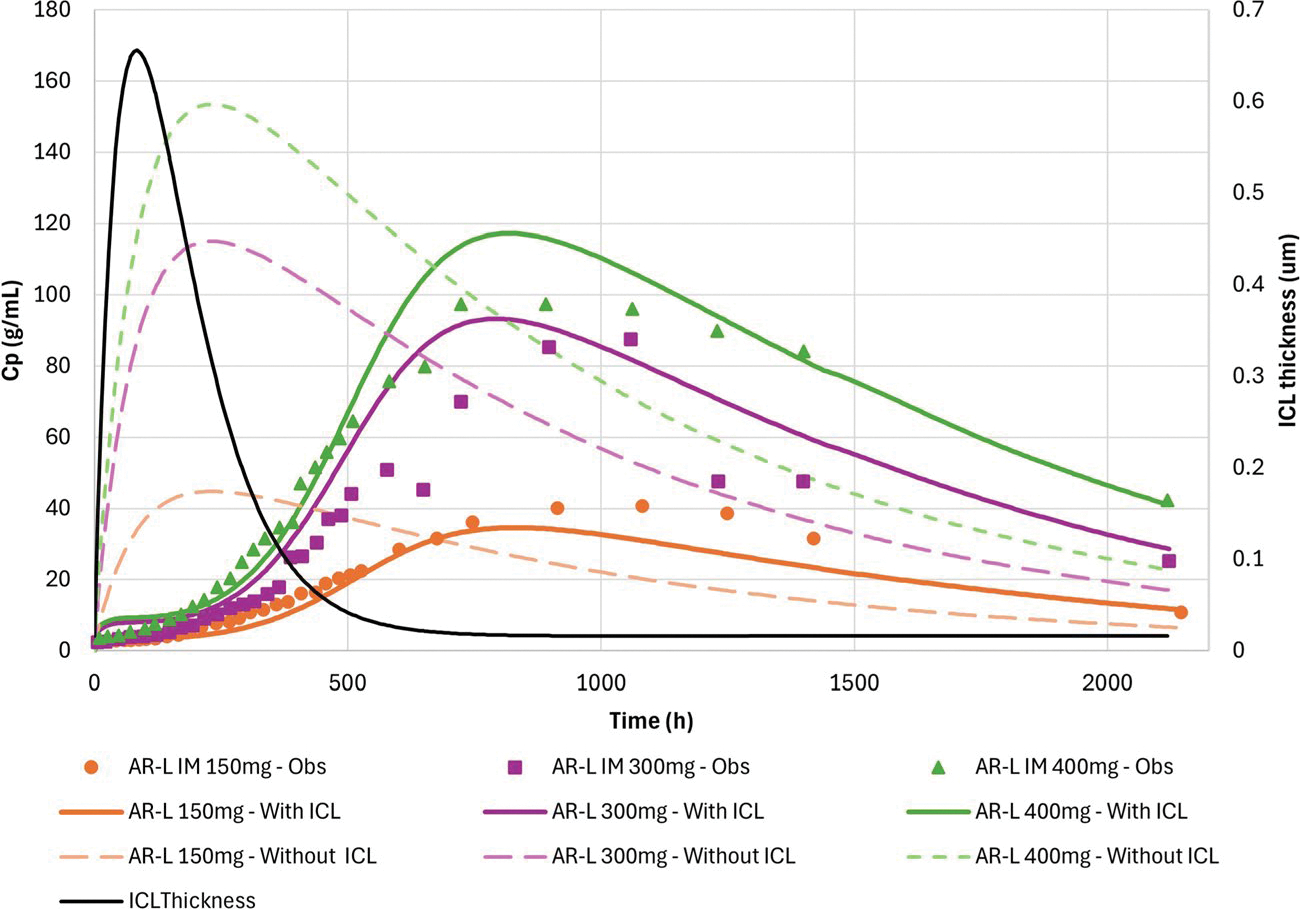
Observed (symbols) and predicted (lines) AR plasma concentration vs time profile after IM administration of aripiprazole lauroxil (AR-L) suspension in humans. Comparison of model with and without the inclusion of Immune cell layer. Left axis: plasma concentration (ng/mL); right axis: ICL thickness (μm)

The calculated IM Kp and f_ut_ for AR-L were 4.28 and 2.25E-06%, respectively. The initial prediction was based on the assumption that the absorption rate is solely dependent on the dissolution of AR-L and partitioning of the dissolved AR-L into the systemic circulation from the IM depot. The simulation results showed a shape mismatch between the simulated curve and observed data and an earlier predicted T_max_ (Fig. 5). The predicted exposure (AUC_0-t_) was in better agreement with the observed values, indicating a discrepancy in the transfer rate and further model adjustments were needed. I.e., predicted C_max_ and AUC_0-t_ for the 150, 300 and 400 mg doses were C_max_ 44.8 ng/mL and AUC_0-t_ 48,270 ng-h/mL; C_max_ 115.11 ng/mL and AUC_0-t_ 123,600 ng-h/mL; C_max_ 153.48 ng/mL and AUC_0-t_ 164,700 ng-h/mL, respectively.

The delayed appearance in systemic circulation after IM administration of AR-L suspension may be attributed to the formation of a dynamic ICL around the injected material (depot). The ICL presents an additional barrier for the dissolved drug to diffuse through before reaching the systemic circulation, explaining the delayed T_max_.

The calculated drug diffusivity and fraction unbound of AR-L in the ICL, and the ICL surface area were 1.12E-6 cm^2^/s, 0.34245% and 2.32 cm^2^, respectively (Eqs. 2, 3 and 4). The ICL parameters A and B (Eq. 1) were 2.09E-6 cm/hr and 0.012 1/hr respectively, which were fitted to the observed PK data for the 400 mg equivalent IM Aristada dose. The same ICL thickness kinetic was used to simulate the other IM Aristada doses. The inclusion of an ICL in the model resulted in adequate predictions (fold errors less than 25%) of the exposure parameters (Table IV) and of the shape of the plasma concentration–time profiles (Fig. 5) for all three dose levels.

### Parameter Sensitivity Analysis

The model without ICL was only sensitive to solubility, particle size and diffusion layer thickness (Supplemental Fig. 4). Even though the model was sensitive to these parameters, varying them was not sufficient to capture the observed data. The only way to properly describe the observed data was through the inclusion of ICL.

**Fig. 6:**
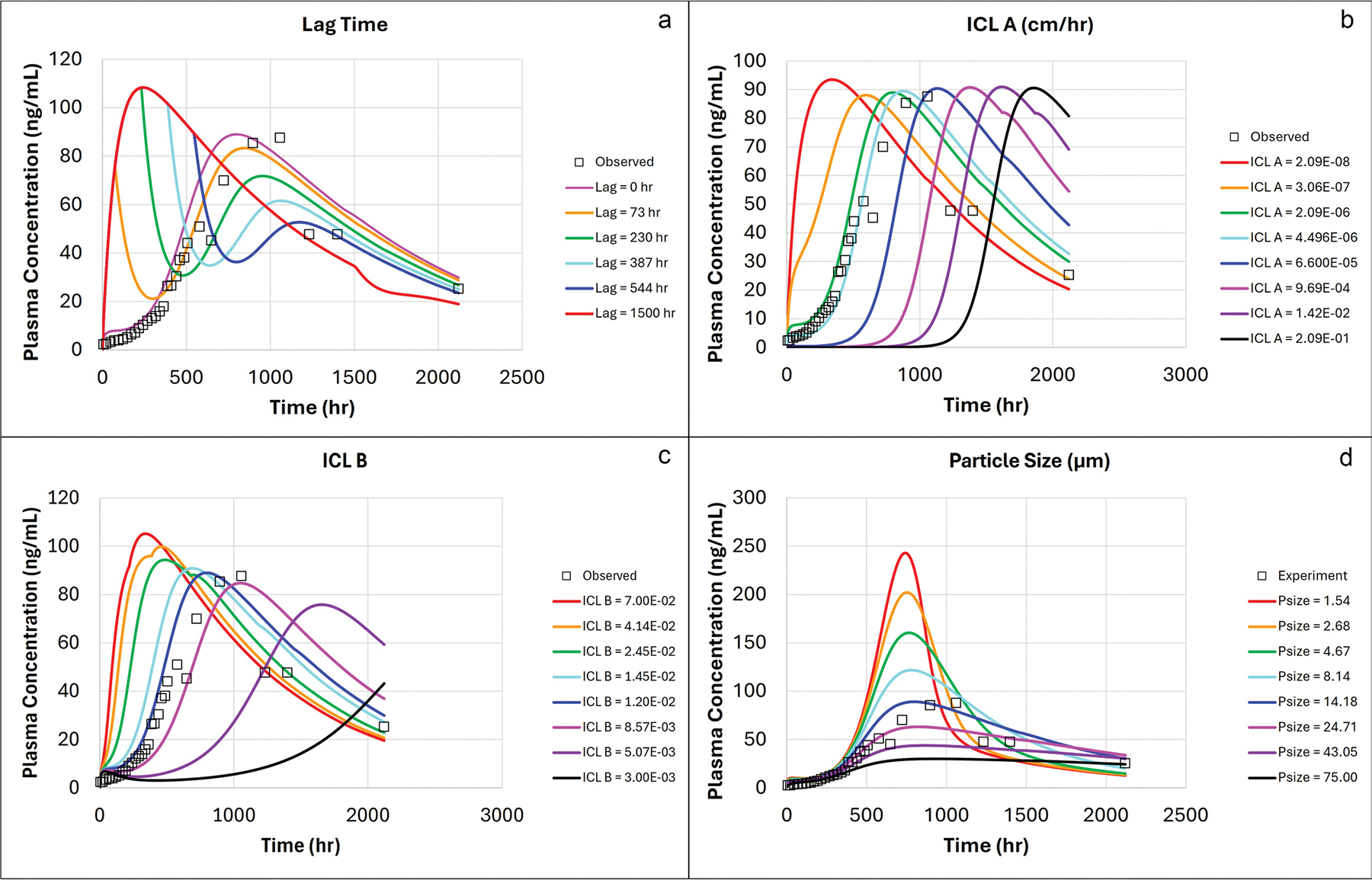
Parameter Sensitivity Analysis on ICL parameters Lag Time (**a**), magnitude (**b**), rate (**c**) and particle size (**d**). Baseline values for each variable: Lag Time = 0 h; Magnitude = 2.09E-06; Rate = 1.2E-02 and Particle Size = 14.18 μm

The impact of lag time (i.e., time elapsed before the onset of ICL) is demonstrated in Fig. 6a. In the absence of ICL, the drug can dissolve and be absorbed unimpeded, resulting in rapid uptake into systemic circulation during the hours that ICL was not present. Lower values of ICL-A parameter will reduce the thickness of the ICL whereas higher values increase the ICL maximum thickness. The impact of ICL-A parameter is shown in Fig. 6b. The impact of ICL-B parameter, which, together with ICL-A, modifies the length of time that the ICL takes to reach maximum thickness, is shown in Fig. 6c. The particle size impact is shown in Fig. 6d. Only after the ICL decay did the particle size play a role, i.e. while ICL was in place (first 300 h) the impact of particle size was minimal, however, after the ICL decay, the smaller the particle size, the higher the exposure.

## Discussion

The skeletal muscle is organized in bundles of fascicles which are composed of bundles of fibers. These bundles are surrounded by a loose connective tissue dominated by an extracellular matrix (ECM). The majority (if not all) of the injected formulation goes into the ECM in the endomysium (true IM depot), perimysium and/or epimysium. Hence, the formulation’s interaction with ECM components and dissemination in that space may be crucial to the drug’s fate after administration (25, 26). Drug release from LAI suspensions is controlled by many parameters, such as the physicochemical properties of the API (e.g., solubility in the fluid at the injection site and accessible surface area) as well as formulation parameters (e.g., excipients, particle size and particle surface properties) (27, 28). The reaction of the tissue to the injected formulation is not yet fully understood (8) and more studies dedicated to *in vivo* depot characterization would be beneficial (6, 29).

In this scenario, PBPK modeling can be valuable in establishing a link between formulation parameters and *in vivo* performance by incorporating the formulation interactions with physiology at the depot site. This work evaluated the use of PBPK modeling informed by *in vitro* formulation characterization (particle size) in a top-down approach to explain the observed PK data by integrating known mechanisms for LAI suspensions.

The systemic disposition model calibrated against oral solution and IV data was validated with several clinical scenarios (Table III). The model was then applied to an IM solution administration. The model was able to capture the overall exposure and profile shape accurately (Fig. 4), even though the C_max_ was slightly overpredicted (fold-error 1.39). Since this was a gluteal injection, part of the dose could have been injected into the subcutaneous space (missed injection site) which would impact the tissue partition coefficient. Jucker et al. examined injection-site variability (among other parameters) for IM cabotegravir LA injections in eight healthy volunteers (24). Even though cabotegravir LA injection was performed under ultrasono-graphic guidance, the depot location was quite variable among the eight participants. One participant received the injection in the retroperitoneal cavity, two participants in the IM tissue, one participant in the subcutaneous tissue, and four participants in both IM and subcutaneous tissues. The authors reported a markedly lower Cmax for the participant who received the injection in the subcutaneous space compared to true IM depot (24).

Another aspect to consider is that the absorption of dissolved compounds follows certain well established physicochemical principles such as the balance between lipophilicity and water solubility. An important aspect of the IM milieu is its pH and restricted environment. Drugs administered as IM solution that do not have good solubility at physiological pH may precipitate at the injection site once the vehicle is absorbed (28). The precipitated particles may redissolve gradually or undergo phagocytosis by macrophages. The phagocytosed particles may be degraded in the acidic lysosomal environment or eventually get released from the macrophages and reach systemic circulation (26). This process was not incorporated in the model since the reported bioavailability of the IM solution was 98% (12). Additionally, given the variability in the observed data in the absorption phase, the IM Kp for AR was kept at the calculated value of 1.84.

The same IM absorption model approach was applied to the AR-L IM suspension administration, specifically Lukacova (default) method for perfusion-limited tissue to calculate the IM Kp and the default GastroPlus v9.5 method to calculate f_ut_ for AR-L. Sanrame et al. described the ideal mechanism of release for N-acyloxymethyl prodrugs such as AR-L (19). In short, rate-limiting dissolution of the prodrug from the depot with subsequent rapid prodrug absorption into systemic circulation and enzymatic cleavage provides efficacious concentrations of the active drug and relatively low prodrug concentration. The authors also described the conversion rate of N-acyloxymethyl prodrugs to the active drug, and the rate of enzymatic cleavage for the lauroyloxymethyl derivative, which is the closest to AR-L, was 20 min. Hence, the systemic prodrug hydrolysis rate was considered to be instantaneous and complete.

The release and absorption of LAI formulations is a complex process as there are many governing mechanisms. It is generally assumed that the drug absorption from IM depots is mainly driven by the drug release rate from the formulation and therefore controlled by properties of the dosage form such as particle size, excipients, stabilizers, etc. While these are of crucial importance, drug dissolution and absorption are also greatly influenced by the local physiology and host’s response to the injected material (9, 29). The predictions, based solely on drug particle size, solubility at the injection site, and depot volume, yielded a significantly earlier T_max_ than observed (Fig. 5), suggesting that an additional mechanism should be taken into account.

As described previously, a local acute inflammatory response to injected foreign material may lead to local oedema, affecting the volume available for dissolution. However, this mechanism was not incorporated in the current model, since delayed drug appearance in systemic circulation was observed. Another inflammatory response that may develop in response to the injected foreign material is characterized as a dense fibrotic capsule formed around the depot composed of mixed inflammatory cells (5, 8, 29). As the inflammation subsides, the fibrotic layer becomes thinner which may result in faster drug appearance in systemic circulation. The type, extent and kinetics of the host response depend on formulation properties, such as particle size and excipients, as well as physicochemical properties of the compound (8, 9, 26). This process was included in the model via an ICL with thickness varying over time, as shown in Fig. 5. The predicted systemic exposure was very low in the first days post injection, representing the largest ICL thickness (~ 0.65 μm at 100 h post injection). As the inflammatory response decreases, the ICL becomes thinner which was accompanied by an increase in the predicted plasma concentration profile (Fig. 5). Since all three doses were the same formulation, the ICL process was assumed to be similar between all dose levels. There is no direct measurement to support this hypothesis at this stage, however, the approach utilized to explain the observed PK data was able to accurately describe the Cp-time profiles across all doses (Table IV).

Research has shown that a large number of macrophages are recruited to the injection site, and they infiltrate the formulation depot and may form multinucleated giant cells (29). Drug particles may be phagocytosed at the periphery of the depot and this may present an important dissolution and absorption mechanism since the uptake of particles into macrophages during the chronic inflammatory phase may serve as secondary depots (7). Hence, the drug release may consist of a combination of interstitial and intracellular dissolution. The intracellular dissolution would be followed by passive permeation from within the macrophages to extracellular compartment in the muscle or to the lymphatic system before reaching systemic circulation (9, 26, 29), with the latter being associated with a delayed appearance in systemic circulation (30). Currently, more data is needed to quantitatively and mechanistically define these processes.

Ho et. al. (5) investigated the effect of drug particle size on *in vivo* pharmacokinetic profiles and local inflammatory responses following IM injection of entecavir-3-palmitate (EV-P) particles in rats. The EV-P crystals with different median diameters (0.8, 2.3, 6.3, 15.3 and 22.6 μm) showed size dependent *in vitro* dissolution profiles under sink conditions. Following IM injection in rat, the pharmacokinetic profile of EV exhibited marked size-dependency with the smaller sizes having higher systemic exposure compared to the larger ones. The particle size dependent pharmacokinetic pattern was also highly associated with histopathological responses at the injection site. The smaller particles presented lower infiltration of inflammatory cells, and thinner fibroblastic bands around depots after 4 weeks. Conversely, larger particle size led to more severe fibrosis, which increased the amount of drug remaining at the injection site over 4 weeks. This, in turn, decreased drug dissolution and systemic exposure (5).

Paquette et. al. (8) evaluated the host reaction to subcutaneous (SC) and IM LAI suspensions of aripiprazole monohydrate (dv50 = 5 μm) and olanzapine pamoate monohydrate (dv50 = 7.4 μm), both compounds from the same class, i.e. atypical antipsychotics. The authors evaluated the histopathology of the IM injection sites in monkeys and the SC injection in rats. A foreign body reaction localized at the injection site was reported with an increased level of proinflammatory cytokines in the acute inflammatory phase followed by a giant cell-free chronic inflammation. Interestingly, the authors reported drug-dependent response both in the histopathology observations and cytokine release at the depot site, which indicates that drugs in the same class may elicit different inflammatory response (8).

The PSA demonstrated the utility of this model for understanding how impactful differences in physiological response and drug product particle size are on the pharmacokinetics of AR-L LAI (Fig. 6). When it comes to formulation design space for a given drug, the variation in inflammatory response would most likely be linked to product parameters such as drug loading, particle size, excipients, etc. While inflammatory response cannot be predicted at this juncture, pre-clinical and/or clinical studies coupled with PBPK modeling could help parametrize some of these parameters. The model can then be applied to conduct sensitivity analysis and this understanding may help with design space questions. For example, Fig. 6D shows that in the first couple weeks after administration, the physiological response (ICL) has more impact on the PK than the particle size distribution since variations in the particle size did not result in significant changes in systemic exposure. The particle size only had an impact after the inflammatory response had decayed. Future studies to elucidate the interplay between the physicochemical properties of compounds, formulations parameters and tissue dynamics at the site of injection are needed to give better insight on formulation *in vivo* performance.

## Conclusion

The drug absorption and PK profile of LAI suspensions are primarily affected by the kinetics of drug dissolution and the local inflammatory response at the injection site. The inflammatory response to the injected solid materials may result in a diffusion barrier at the injection site, resulting in delayed drug release and absorption. Utilizing a time-dependent change in ICL thickness across all dose levels resulted in reasonable predictions of AR PK profiles following IM administration of multiple strengths of the AR-L suspension. This shows the utility of PBPK model in mechanistically describing the *in vivo* performance of LAIs. Predicting the exposure of LAIs is challenging given the high variability and lack of available data to accurately parameterize the model, hence further studies are needed to validate and refine this approach.

## Supplementary Material

Supplemental Material

**Supplementary Information** The online version contains supplementary material available at https://doi.org/10.1208/s12248-025-01171-1.

## Figures and Tables

**Table I.** Human PK Data Used for Model Development and Validation

Compound and Dosing	Use in the model	Reference

AR Oral solution (15, 20 and 30 mg)	PK model development	(11, 12)
AR IV infusion (2 mg)	PK model verification	
AR Oral solution (5 and 10 mg)	PK model validation	(11)
AR Oral tablet (5, 10, 15 and 30 mg)		
AR IM solution (5 mg)	PK model validation/IM absorption model development	(12)
AR-L IM suspension (400 mg equivalent)	ICL model development	(13)
AR-L IM suspension (150 and 300 mg equivalent)	ICL model validation	(13)

a thorough literature search was conducted to identify the clinically observed data, however this was not a systemic review

**Table II.** Summary of PBPK input parameters for AR-L and AR

Input Parameter	Value		Rationale/Reference(s)
Physicochemical Properties	AR-L	AR	

Molecular mass (g/mol)	660.73	448.38	APv11.2
Log P	9.8	5.2	APv11.2
pKa	7.47 (base)	7.6 (base)	AR-L: APv11.2 AR: (14)
Precipitation time (s)	900	900	GastroPlus default^†^
Aqueous solubility (mg/mL)	0.0003 (@ pH 7)	0.004 (@ pH 7)	AR-L: (15) AR: (16)
Solubility Factor	10200	660.92	APv11.2
Blood-to-plasma ratio (R_bp_)	0.64	0.61	AR-L: APv11.2 AR: (14)
F_u_, plasma (%)	0.2	3.17	AR-L: APv11.2 AR: (14)
**Oral Absorption**			
Human effective jejunal permeability (P_eff_)(×10^−4^ cm/s)		3.18	APv11.2
Bile salt solubilization ratio		252000	(17)
Dissolution model—oral		Johnson model	Default
Particle radius (μm)		22	Fitted to plasma concentration data across all doses
**Disposition**			
Compartmental PK		V_c_: 1.5825 L/kg CL_H_: 0.03028 L/h/kg k_12_: 0.22832 h^−1^ k_21_: 0.17345 h^−1^ k_13_: 0.01108 h^−1^ k_31_: 0.001911 h^−1^	Fitted and verified using IV and oral suspension data (11, 12)
**Intramuscular**			
**Injection Volume (mL)**			
5 mg AR		0.67	(12)
150 mg AR equivalent dose	0.805		(13)
300 mg AR equivalent dose	1.6		
400 mg AR equivalent dose	2.13		
**Effective Depot Volume (mL)** ^‡^			Calculated using default method within GastroPlus
5 mg AR		5.7	
150 mg AR equivalent dose	6.8		
300 mg AR equivalent dose	13.6		
400 mg AR equivalent dose	18.1		
Percent Unbound in Muscle Tissue (%)*	2.25E-06	0.12	Default method
Muscle/Plasma partition coefficient (K_p_)*	4.28	1.84	Lukacova (default) method for perfusion limited model
Unstirred water layer thickness (μm)	65		Fitted to 400 mg eq. PK data
Dissolution model	Johnson model		Default
Particle radius (μm) ± Standard deviation	14.18 ± 7.4		Fitted to D10, D50, D90 measured data
**Immune Cell Layer**			
Lag Time (hr)	0		Fitted to 400 mg eq. PK data (12)
Parameter A (cm/hr)	2.09E-06		
Parameter B (1/hr)	0.012		
Diffusivity (cm^2^/s)	1.12E-06		Calculated within GastroPlus
Percent Unbound (%) Surface Area (cm^2^)	0.34245		Calculated within GastroPlus
150 mg AR equivalent Dose	1.21		Estimated based on injected dose assuming spherical depot shape (Eq. 4)
300 mg AR equivalent Dose	1.92		
400 mg AR equivalent Dose	2.32		

† A parameter sensitivity analysis was conducted to assess the impact of precipitation time on fraction absorbed (Fa) across the doses. Precipitation time did not have an impact on Fa, hence the default value was used

* Both Kp and f_ut_ calculations are based on drug (logP, pKa, adjusted fraction unbound in plasma (Fup), and blood/plasma concentration ratio (Rbp)) and physiological (tissue composition) properties

APv11.2: ADMET Predictor version 11.2

‡ Effective depot volume is calculated as injection volume/extracellular volume fraction, which in GastroPlus is defined as 0.118.

**Table III.** Comparison of C_max_ and AUC_0-t_ fold-errors after AR IV and oral administration

	Cmax (ng/mL):			AUC 0-t (ng-h/mL):			Use in the model
	Observed	Simulated	FE	Observed	Simulated	FE	

IV 2 mg infusion	16.71	14.63	0.88	522.16	547.95	1.05	Model verification
Oral Solution 5 mg	23.96	25.87	1.08	1598.20	1580.90	0.99	Model validation
Oral Solution 10 mg	46.04	51.75	1.12	3293.30	3161.60	0.96	Model validation
Oral Solution 15 mg	72.92	77.62	1.06	5146.10	4742.40	0.92	Model development
Oral solution 20 mg	88.74	103.50	1.17	6626.80	6323.60	0.95	Model development
Oral Solution 30 mg	126.62	155.23	1.23	9879.30	9485.40	0.96	Model development
Oral Tablet 5 mg	22.89	19.87	0.87	1664.90	1473.30	0.88	Model validation
Oral Tablet 10 mg	39.58	39.60	1.00	2832.60	2942.70	1.04	Model validation
Oral Tablet 15 mg	55.20	59.19	1.07	4306.30	4408.10	1.02	Model validation
Oral Tablet 30 mg	99.36	117.14	1.18	8780.60	8778.90	1.00	Model validation

FE = fold-error (simulated/observed).

**Table IV.** Comparison of C_max_, AUC_0-t_ and T_max_ fold-errors after AR-L IM administration

	C_max_ (ng/mL):			AUC_0-t_ (ng-h/mL):			T_max_ (h)		
	Obs	Sim	FE	Obs	Sim	FE	Obs	Sim	FE

IM suspension 150 mg	40.66	34.63	0.85	52,810	43,140	0.82	1081.9	829.17	0.77
IM suspension 300 mg	87.61	88.99	1.02	91,910	110,600	1.20	1058.2	798.83	0.75
IM suspension 400 mg	97.39	117.29	1.20	138,100	146,200	1.06	723.26	818.88	1.13

Obs: Observed; Sim: Simulated; FE: fold-error (simulated/observed).
