# Supplementary material for: Mechanistic Modeling of Intramuscular Administration of a Long-acting Injectable Accounting for Tissue Response At the Depot Site
Source: AAPS J. Author manuscript; Available in PMC 2025 Nov 6. (PMC12591097; doi:10.1208/s12248-025-01171-1)

Supplemental materials

**Supplemental Figure 1.** Graphical representations of TGA and DSC profiles of aripiprazole lauroxil


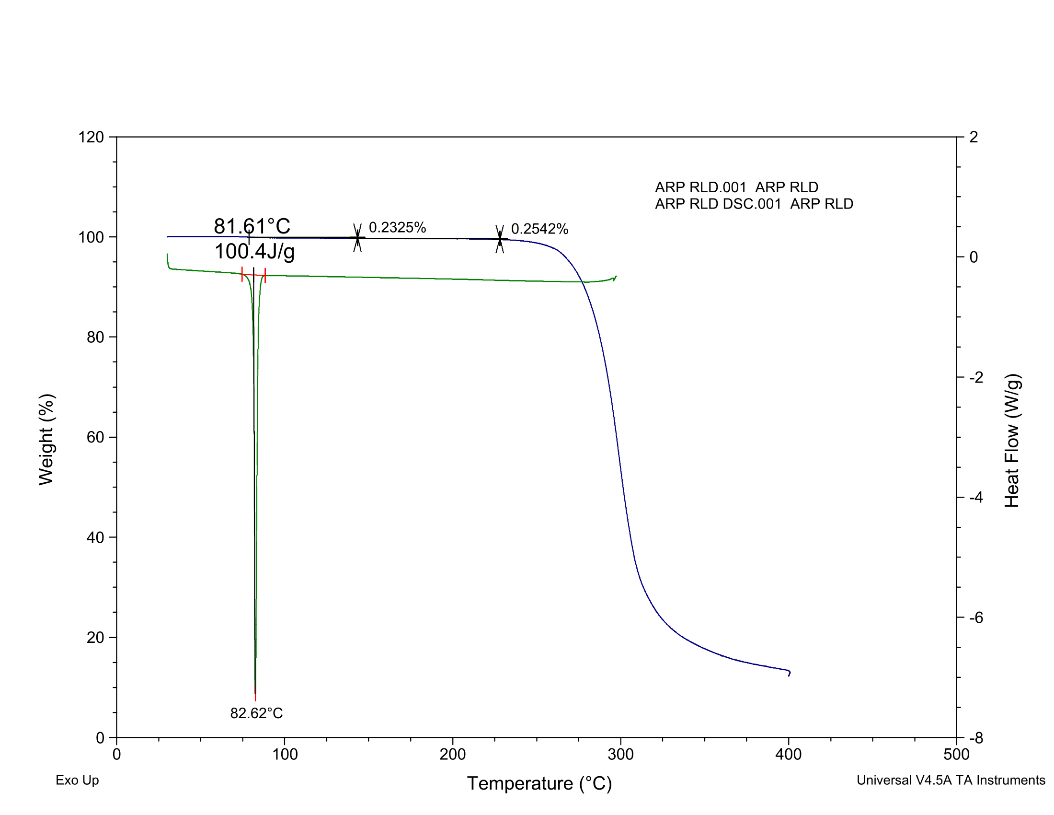


**Supplemental Figure 2.** Graphical representation of PXRD pattern of Aripiprazole Lauroxil


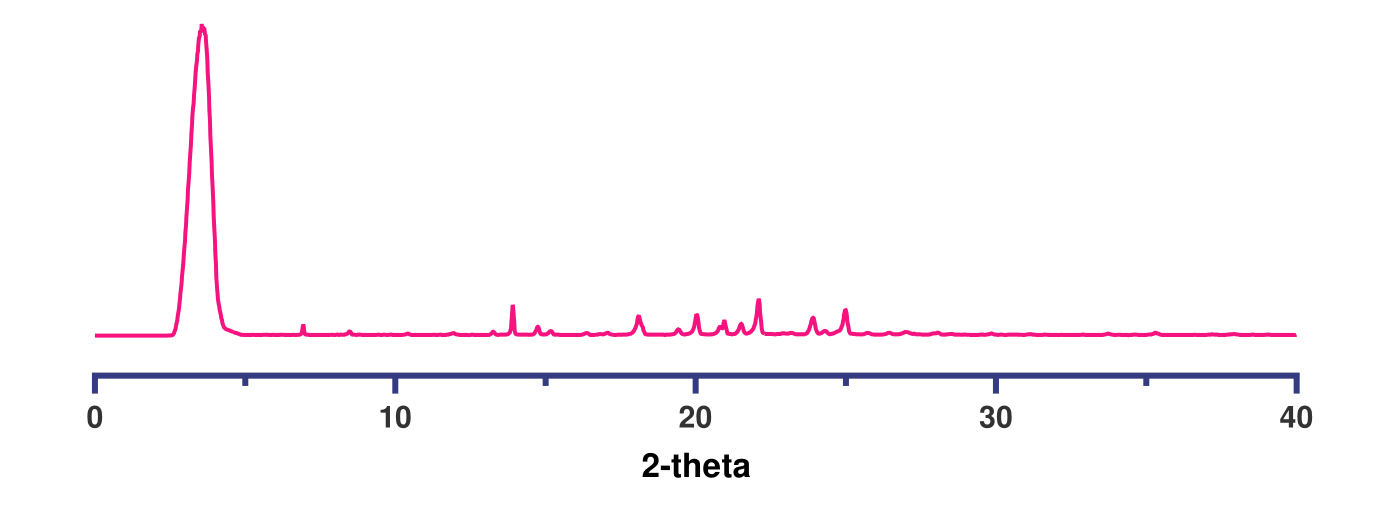


**Supplemental Figure 3.** Microscopy imaging of Aristada® containing aripiprazole lauroxil particles at 50X objective and a 100 µ scale. Imaging performed using a Keyance polarized light microscope equipped with LINK imaging software.


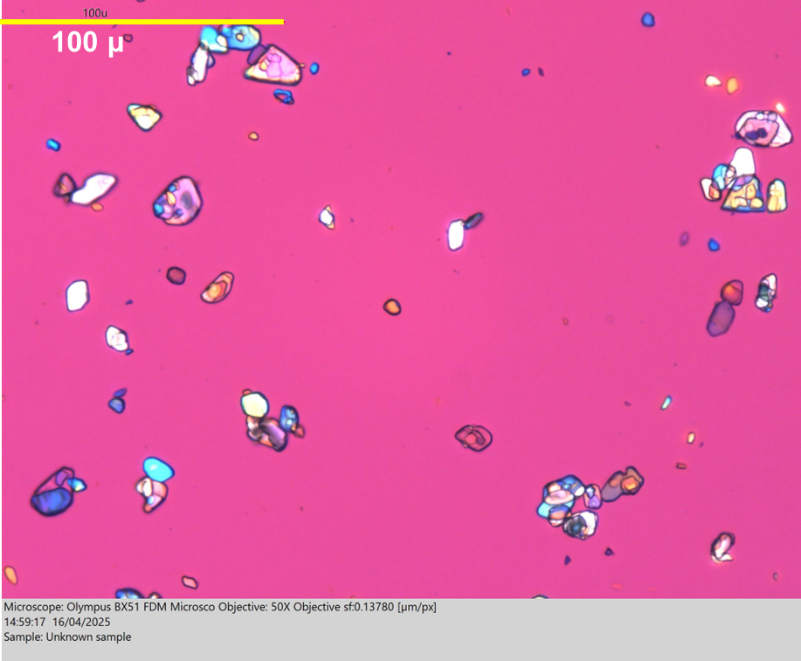


**Supplemental Table 1.** Solubility data of aripiprazole lauroxil in different media

| **Media** | **Solubility (µg/mL)** |
| --- | --- |
| 50 mM PBS pH 3 | - |
| 50 mM acetate buffer pH 4 | - |
| 50 mM PBS pH 6 | - |
| 50 mM PBS pH 7.4 | - |
| 50 mM tris buffer pH 9 | - |
| 0.1% SDS in water | 0.54 ± 0.03 |
| 0.25% SDS in water | 0.56 ± 0.01 |
| 0.5% SDS in water | 18.05 ± 1.13 |
| 1% SDS in water | 36.87 ± 2.77 |
| 0.1 % Tween 20 in water | - |
| 0.25% Tween 20 in water | 2.85 ± 0.16 |
| 0.5% Tween 20 in water | 19.60 ± 3.89 |
| 1% Tween 20 in water | 47.31 ± 7.91 |
| Suspending media | 7.27± 0.21 |

**Supplemental figure 4.** Parameter Sensitivity Analysis on model variables Solubility (A), Diffusion layer thickness (B), and Particle size (C). Baseline values for each variable: Solubility = 3E-4 mg/mL; Diffusion layer thickness = 65 µm; and Particle Size = 14.18µm.


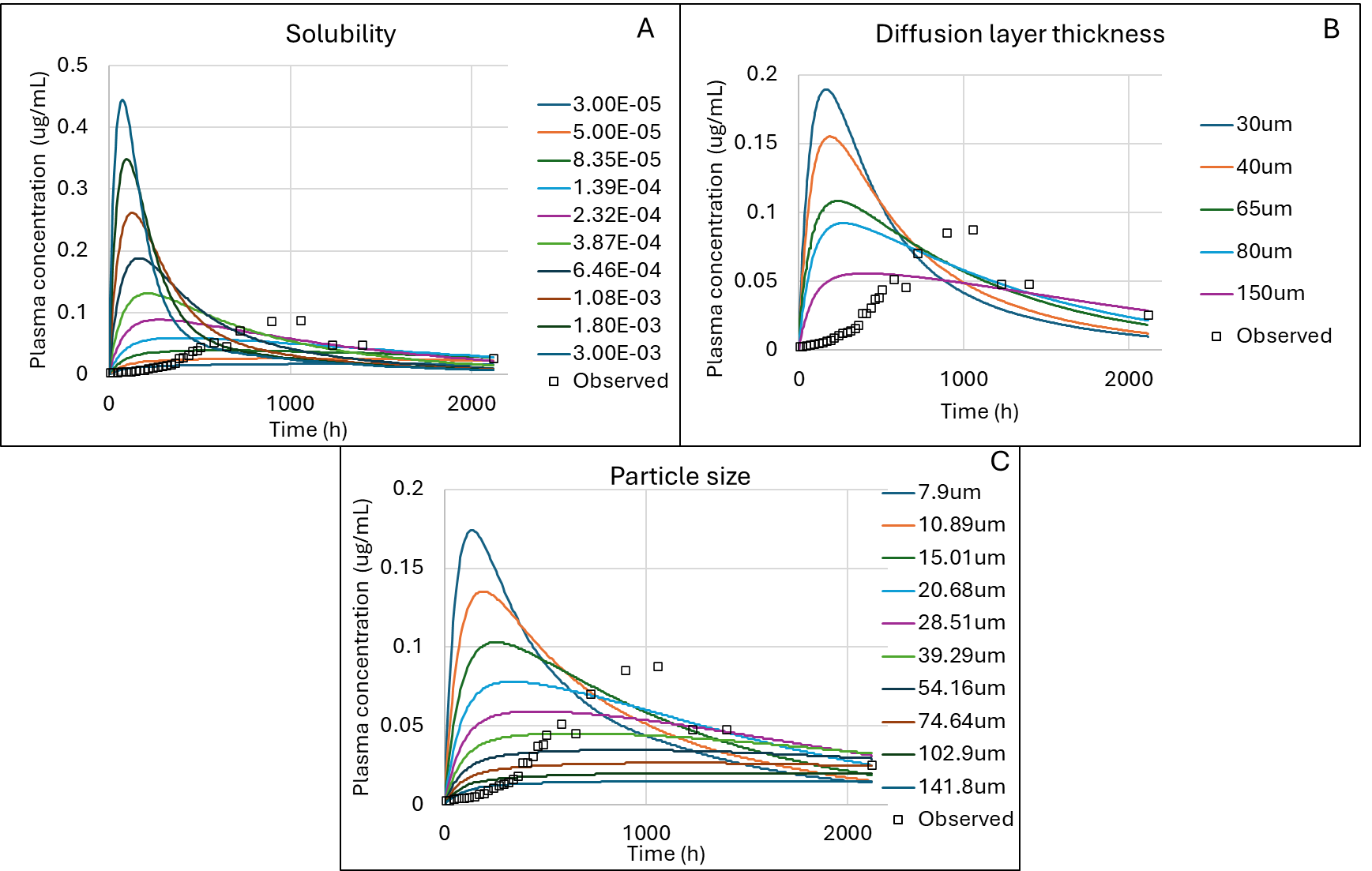

Supplement: Supplemental Material [file NIHMS2120713-supplement-Supplemental_Material.docx]
